# Using Time-out for Child Conduct Problems in the Context of Trauma and Adversity: A Nonrandomized Controlled Trial

**DOI:** 10.1001/jamanetworkopen.2022.29726

**Published:** 2022-09-01

**Authors:** Alex C. Roach, Meryn Lechowicz, Yu Yiu, Antonio Mendoza Diaz, David Hawes, Mark R. Dadds

**Affiliations:** 1 School of Psychology, Faculty of Science, University of Sydney, Sydney, New South Wales, Australia; 2 Department of Psychology, Faculty of Humanities and Social Sciences, The University of Bath, Bath, United Kingdom; 3 Discipline of Psychiatry and Mental Health, Faculty of Medicine, University of New South Wales, Sydney, New South Wales, Australia

## Abstract

**Importance:**

Exposure to adverse childhood experiences substantially increases the risk of chronic health problems. Originally designed to treat child conduct problems, parent management training programs have been shown to be effective in preventing children from being exposed to further adversity and supporting children’s recovery from adversity; however, there are increasing concerns that a core component of these programs, the discipline strategy time-out, may be harmful for children with a history of exposure to adversity.

**Objective:**

To investigate the comparative benefits and potential harms to children exposed to adversity that are associated with parenting programs that include time-out.

**Design, Setting, and Participants:**

This nonrandomized waiting list–controlled clinical study was conducted at a specialist clinic for the treatment of conduct problems in Sydney, Australia. The self-referred sample included children with conduct problems and their caregivers. Eligibility was confirmed through clinician-administered interviews. Data were collected between February 14, 2018, and February 1, 2021.

**Interventions:**

Caregivers participated in a 10-session, social learning–based parent management training program. Caregivers were provided with parenting strategies aimed at encouraging desired behaviors through effective reinforcement and managing misbehavior through consistent limit setting, including the use of time-outs.

**Main Outcomes and Measures:**

The primary outcome was the parent-reported Strengths and Difficulties Questionnaire score, and secondary outcomes included subscale scores from the clinician-administered Diagnostic Interview Schedule for Children, Adolescents, and Parents. Multi-informant measures of child adversity were collected using the parent-reported Adverse Life Experiences Scale and the clinician-rated Maltreatment Index.

**Results:**

A total of 205 children were included in analysis (156 in the full intervention and 49 in the control condition; 158 boys [77.1%]; mean [SD] age, 5.6 [1.8] years [range, 2-9 years]). Compared with children with low adversity exposure, children with high adversity exposure showed greater reductions in the Strengths and Difficulties Questionnaire score from baseline (mean difference, 3.46 [95% CI, 1.51-5.41]; *P* < .001) to after treatment (mean difference, 1.49 [95% CI, −0.46 to 3.44]; *P* = .13) and in the internalizing symptom subscale score (baseline mean difference, 1.00 [95% CI, −2,00 to 0.00]; *P* = .50; posttreatment mean difference, 0.06 [95% CI, −0.82 to 0.94]; *P* = .90). No significant differences in the externalizing symptom subscale score were found.

**Conclusions and Relevance:**

In this nonrandomized clinical study, children with high exposure to adversity experienced equivalent, if not greater, benefits associated with parenting programs that include time-out compared with children with low exposure to adversity. Results suggest that time-out was an effective component of parenting programs for children exposed to adversity.

**Trial Registration:**

anzctr.org.au Identifier: ACTRN12617001472369

## Key Points

**Question:**

Are parenting programs that include time-out less effective or even harmful for children exposed to adverse childhood experiences?

**Findings:**

In this nonrandomized clinical trial of 205 families, children with conduct problems and high adversity exposure experienced equivalent, if not greater, outcomes, measured by the parent-reported Strengths and Difficulties Questionnaire, after a parenting program that included time-out, compared with children with low adversity exposure.

**Meaning:**

This study suggests that, despite concerns that time-out is contraindicated for children who have experienced adversity, parenting programs that include time-out appear to be beneficial for children with or without adversity exposure for management of emotional and behavioral difficulties.

## Teaser

This nonrandomized controlled trial investigates the comparative benefits and potential iatrogenic harms to children exposed to adversity that are associated with parenting programs that include time-out.

## Introduction

Exposure to adverse childhood experiences (ACEs) is a major public health concern, posing substantial risk for chronic mental and physical health problems.^1,2^ ACEs include experiences of maltreatment, household dysfunction, minority adversities, and stressful life events, with cumulative adversity and caregiver perpetration conferring greater risk for negative outcomes.^3,4^ Epidemiologic studies show that ACEs are common^5,6,7^ and account for nearly 30% of childhood mental health (CMH) disorders.^8^ Exposure to an ACE often occurs in the context of hostile family dynamics, violent discipline techniques, and the absence of stable, nurturing primary attachments.^9,10,11^ Conversely, effective parenting boosts resilience and mitigates the mental health consequences of ACEs.^12,13^ Caregiver actions are considered a modifiable risk factor for exposure to and recovery from ACEs, making programs that target the quality of parenting an important focus for prevention and intervention.

There is growing evidence that parent management training (PMT), an evidence-based intervention for childhood conduct problems,^14^ is an effective mental health response to ACE exposure. Grounded in social learning theory,^15^ PMT focuses on enhancing parenting behaviors and reducing “coercive cycles” within parent-child dyads^16^ to provide children with responsive, consistent, and affirming family environments. The application of PMT to child welfare populations has been associated with reductions in caregiver-perpetrated physical abuse^17,18,19^ and improvements in CMH.^20,21,22,23^ Despite this, core PMT strategies, such as “time-out” (TO), have been criticized in recent years for their use among children with exposure to ACEs.^24^

Based on operant conditioning,^25^ TO is a core component of PMT programs associated with larger effect sizes in efficacy studies.^26^ Time-out functions to set healthy boundaries on children’s behavior while reducing the risk of physical punishment and unintentional reinforcement of undesired behaviors through excessive parental attention. Procedurally, TO involves temporarily placing a child in a setting with minimal reinforcement (eg, chair in hallway) in response to misbehavior (eg, noncompliance or physical aggression), in conjunction with ample positive parent-child interactions during periods of nonproblem behavior.^24^ Despite evidence that caregivers who use TO show reductions in abusive parenting responses,^27,28,29^ there are concerns that TO is itself a form of ACE exposure.^30^

Critics fear that TO is experienced as abandonment and, consequently, ruptures attachment bonds, disrupts developing nervous systems, and retraumatizes children by triggering recollections of maltreatment.^31^ There are also criticisms that TO gains compliance through fear^32^ and that children with a history of ACE exposure are especially vulnerable to potential harm from TO owing to preexisting attachment and neurobiological deficits.^33^ Concerns surrounding TO have been largely due to misleading and unsubstantiated media releases,^30,34^ widespread misinformation, and inaccurate use of TO,^35,36^ resulting in an increasing negative view of the evidence-based strategy.^37^

To our knowledge, there is currently no evidence to suggest that TO is harmful for children with exposure to ACEs; however, there is also limited research comparing how children with and children without ACE exposure respond to PMT programs that include TO (ie, PMT-TO programs). Although proposed concerns about TO would not preclude children benefiting from other PMT components, TO may diminish therapeutic outcomes to a greater degree for children exposed to ACEs, as exacerbated attachment and emotion deficits are known to increase CMH problems.^38,39,40^ Earlier investigations found that PMT-TO programs were equally effective for children with and children without ACE exposure, as determined by child protection reports.^41,42^ These studies did not account for all ACEs, including emotional abuse and neglect, which rarely reach the threshold for a mandatory report but may be especially relevant in predisposing children to experiencing TO as a form of rejection.

The present study addressed this gap by capturing a broad range of ACEs using quantitative measures across multiple informants. Several study hypotheses were developed: (1) all children engaged in a PMT-TO program will experience significant improvements in CMH symptoms compared with those on the waiting list; (2) children with high ACE exposure will be less responsive to a PMT-TO program; and (3) if fear of TO is associated with behavior change, internalizing symptoms are expected to increase after the PMT-TO program, with more pronounced increases for children with high ACE exposure, owing to heightened hypervigilance to threat cues.^1^

## Methods

### Study Design and Setting

This study included a nonrandomized intervention group, a waiting list–controlled group, and groups with high or low adversity exposure (study protocol in Supplement 1). Participants were self-referred to the Child Behavior Research Clinic in Sydney, Australia, between February 14, 2018, and February 1, 2021. Approval for this study was granted by the University of Sydney human ethics committee, and written informed consent was obtained from caregivers at the initial assessment. The Transparent Reporting of Evaluations With Nonrandomized Designs (TREND) reporting guideline^43^ was followed. There were no substantive adverse events or deviations from the study protocol.

### Participants

The sample included 205 children (47 girls and 158 boys), along with caregivers (206 mothers, 177 fathers, and 2 other caregivers) and educators (N = 174). Participants were children aged 2 to 9 years presenting with oppositional defiant disorder or conduct disorder. Children with comorbid attention-deficit/hyperactivity disorder, level 1 autism spectrum disorder, or internalizing disorder presentations were also eligible. Exclusion criteria included major neurologic or physical illness or developmental disability, concurrent engagement in a parenting program, or current child-related legal proceedings. Diagnostic ratings were based on the *Diagnostic and Statistical Manual of Mental Disorders* (Fifth Edition) (*DSM-5*) criteria.^44^ The Table outlines the sociodemographic characteristics of the sample.

**Table.** Demographic Characteristics of Participants and Baseline Variables

Variable	Participants, No. (%)			
	Total (N = 205)	Intervention (n = 156)		Waiting list (n = 49)
		Low adversity (n = 122)	High adversity (n = 34)	
Child age, mean (SD), y	5.6 (1.8)	5.4 (1.7)	6.4 (1.6)	5.5 (1.8)
Child sex				
Female	47 (22.9)	28 (23.0)	10 (29.4)	9 (18.4)
Male	158 (77.1)	94 (77.0)	24 (70.6)	40 (81.6)
Primary caregiver’s age, mean (SD), y	40.2 (4.7)	40.1 (4.6)	41.2 (5.7)	39.8 (4.3)
Participant’s relationship status				
Sole caregiver	34 (16.6)	18 (14.8)	7 (20.6)	9 (18.4)
Married or de facto	155 (75.6)	96 (78.7)	22 (64.7)	38 (77.6)
Divorced or separated	14 (6.8)	7 (5.7)	5 (14.7)	2 (4.1)
Primary caregiver’s educational level				
Year 12 or below	11 (5.4)	5 (4.1)	2 (5.9)	4 (8.2)
TAFE, diploma, or certificate	47 (22.9)	30 (24.6)	8 (23.5)	9 (18.4)
Undergraduate degree	74 (36.1)	47 (38.5)	12 (35.3)	15 (30.6)
Postgraduate degree	73 (35.6)	40 (32.8)	12 (35.3)	21 (42.9)
No. of sessions, mean (SD)	10.7 (2.6)	10.4 (2.4)	10.5 (3.5)	11.4 (2.3)
Comorbid *DSM-5* diagnoses				
ADHD	53 (25.9)	36 (29.5)	11 (32.4)	6 (12.2)
ASD	7 (3.4)	4 (3.3)	2 (5.9)	1 (2.0)
DASS-21 score, mean (SD)				
Depression	2.3 (3.5)	2.3 (3.5)	2.7 (4.0)	2.3 (3.2)
Anxiety	1.6 (2.5)	1.8 (2.8)	1.7 (2.4)	1.1 (1.7)
Stress	6.0 (4.1)	5.8 (3.8)	7.3 (4.9)	5.7 (4.3)

Abbreviations: ADHD, attention-deficit/hyperactivity disorder; ASD, autism spectrum disorder; DASS-21, 21-item Depression Anxiety Stress Scale; *DSM-5*, *Diagnostic and Statistical Manual of Mental Disorders* (Fifth Edition); TAFE, technical and further education.

### Procedure

Assessment data were collected via interviews and online surveys from caregivers at a maximum of 3 time points. A subsample of participants referred to the clinic during periods of extended waiting times (approximately 12 weeks) completed a preliminary assessment, forming the waiting list–controlled group of the study. The pretreatment assessment was completed by the treating clinician, and the posttreatment assessment was completed by a third independent clinician who was unaware of the child’s previous ACE exposure. A Consolidated Standards of Reporting Trials (CONSORT) diagram^45^ describing the flow of participants is provided (eFigure in Supplement 2).

### Parenting Intervention

Participants received the Integrated Family Intervention for Child Conduct Problems,^46^ a social learning–based PMT-TO program with a strong evidence base in the treatment of child conduct problems.^47,48^ The program aimed to reduce child conduct problems by providing parents and caregivers with strategies to effectively reinforce desirable behavior and manage misbehavior (eg, noncompliance or aggression) through consistent and responsive limit setting, which included the use of TO in accordance with evidence-based parameters.^24,46^ Additional intervention modules targeting wider systemic issues (eg, partner conflict) and comorbid child symptoms (eg, anxiety or sleep problems) were included as needed. Parents and caregivers completed approximately 10 weekly, 1-hour individual sessions delivered in person and/or via telehealth by a trained psychologist.

### Measures

#### Primary Outcome: Strengths and Difficulties Questionnaire Score

The Strengths and Difficulties Questionnaire (SDQ) is a 25-item, parental-report measure of children’s psychopathologic symptoms.^49^ The SDQ assesses emotional symptoms, conduct problems, hyperactivity-inattention, and peer problems. Using a 3-point Likert scale (where 0 indicates “not true,” 1 indicates “sometimes true,” and 2 indicates “certainly true”), participants indicated how much the target characteristic applied to their child. The 4 domains were summed to compute a total SDQ score (α = .77). Only the designated primary caregiver was included in analyses to ensure independence of measures.

#### Secondary Outcome: Diagnostic Interview Schedule for Children, Adolescents, and Parents, 5th Edition, Score

The Diagnostic Interview Schedule for Children, Adolescents, and Parents, 5th Edition (DISCAP-V),^50^ is a semistructured interview for assessing common childhood disorders based on the *DSM-5*. Clinicians administer the DISCAP-V with caregivers and assign severity ratings based on a 6-point Likert scale (where 1 indicates minimal impairment and 6 indicates very severe impairment), with 4 equated to meeting diagnostic criteria. There was evidence of strong interdiagnostician agreement for primary (Cohen κ = 0.88), secondary (Cohen κ = 0.78), and tertiary (Cohen κ = 0.63) diagnoses. Externalizing disorders (eg, oppositional defiant disorder or conduct disorder) and internalizing disorders (eg, anxiety or depression disorders) subscale scores were included as secondary outcome measures.

#### Adverse Life Experiences Scale

The Adverse Life Experiences Scale is a 23-item measure of adversity that has demonstrated good reliability and validity.^6^ The Adverse Life Experiences Scale was developed as an extension of the original 10-item ACE survey to capture a broader range of adverse experiences (eg, peer victimization or minority adversity). Items were endorsed on a dichotomous (yes or no) scale and summed (α = .64). Consistent with the wider ACE literature,^4^ high (ACE score ≥4; top 10% of the sample) and low (ACE score ≤3) ACE groups were created for bivariate analyses.

#### Maltreatment Index

The Maltreatment Index is based on the Maltreatment Classification System.^51^ The scale uses a 4-point Likert scale (where 0 indicates never; 1, a little bit; 2, a fair bit; and 3, all the time). Clinicians rate the veracity of 3 subtypes of maltreatment perpetrated by a trusted adult: emotional abuse, physical abuse, and neglect. A specific focus on maltreatment was included because this ACE subtype is most strongly associated with severe CMH consequences,^52^ which may make children with exposure to maltreatment especially vulnerable to the speculated harms of TO. A total maltreatment score was created by taking the maximum score of the 3 Maltreatment Index items, after which low maltreatment (Maltreatment Index total score ≤1) and high maltreatment groups (Maltreatment Index total score ≥2) were dummy coded.

#### Depression Anxiety Stress Scale

The Depression Anxiety Stress Scale^53^ is a 21-item questionnaire with demonstrated validity and reliability^54^ that assessed 3 domains of adult psychopathologic conditions: depression, anxiety, and stress (α = .92). Although parental mental health itself can be considered an ACE, the Depression Anxiety Stress Scale was included as a covariate to account for acute stressors that participants may have experienced because of the COVID-19 pandemic.

### Statistical Analysis

Analyses were conducted using SPSS, version 26 (IBM Corp) from August 2021 to January 2022.^55^ Preliminary analyses used independent-samples *t* tests and χ^2^ tests of independence to explore the baseline comparability of sample subgroups, and a linear model was conducted to identify factors associated with treatment outcomes. Variables found to be significantly associated with subgroup differences or treatment outcomes were entered as covariates in further analyses.

Relevant statistical assumptions were checked prior to each analysis, and 2-sided *P* ≤ .05 was considered significant. A sensitivity power analysis using G*Power, version 3.1.9.7 (UC Regents)^56^ determined that there was adequate sensitivity to detect even small effects with a power level greater than 0.80, based on the sample sizes used in the main study analysis, 2-sided hypothesis tests, and an α level of .05. Q-Q plots were inspected and confirmed the normal distribution of the data. Further tests of multicollinearity, homogeneity of variance, and covariance matrices were satisfied, and thus the data were considered acceptable for parametric analysis.^57^

A mixed between-within analysis of variance with time as the within factor (differences between preliminary assessment and preassessment and between preassessment and postassessment) and condition (intervention group and waiting list group) as the between factor was conducted to compare waiting list and intervention outcomes. A mixed analysis of variance with time as the within factor (differences between preassessment and postassessment on the SDQ and DISCAP-V) and ACEs (high ACEs and low ACEs) as the between factor were conducted with the intervention group to examine the association of adversity with children’s responsiveness to the PMT-TO program.

## Results

A total of 205 children were included in the analysis (156 in the full intervention and 49 in the control condition; 158 boys [77.1%]; mean [SD] age, 5.6 [1.8] years [range, 2-9 years]). The baseline characteristics of all of the participants and their subgroups are given in the Table. There were no significant differences found between the intervention and waiting list groups across demographic and baseline variables. Children in the high adversity group were slightly older than children in the low adversity group (mean [SD], 6.4 [1.6] vs 5.4 [1.7] years). Rates of participant noncompletion were equivalent between the intervention and waiting list groups, and there were no significant differences between completers and noncompleters. A linear model with posttreatment SDQ scores included as the dependent variable identified several variables associated with treatment outcomes, including treatment dose, comorbid attention-deficit/hyperactivity disorder, and caregiver stress and anxiety, that were included as covariates along with child age in further analysis.

**Figure 1.**
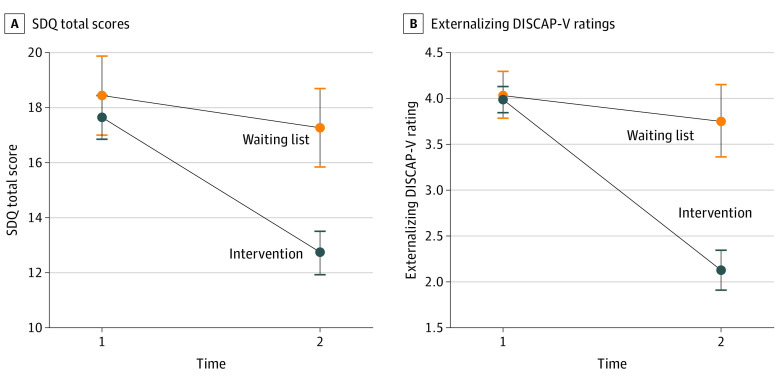
Effectiveness of Parent Management Training Including Time-out Intervention Plots comparing intervention and control group Strengths and Difficulties Questionnaire (SDQ) and externalizing Diagnostic Interview Schedule for Children, Adolescents, and Parents, 5th Edition (DISCAP-V) mean ratings at time 1 and time 2 for outcomes with significant time × group interactions. Error bars indicate 95% CIs.

A significant interaction effect of time and condition (*F*_1,198_ = 24.51; *P* < .001) showed that, while the intervention and waiting list groups had similar baseline scores, the intervention group had lower posttreatment scores on both the SDQ (mean baseline difference, 0.79 [95% CI, −0.86 to 2.45]; *P* = .35; mean posttreatment difference, 4.55 [95% CI, 2.91-6.20]; *P* < .001) and the externalizing DISCAP-V (mean baseline difference, 0.05 [95% CI, −0.25 to 0.35]; *P* = .74; mean posttreatment difference, 1.63 [95% CI, 1.18-2.08]; *P* < .001) compared with the waiting list group (Figure 1; eTable 1 in Supplement 2). Although the present study was not aiming to evaluate the effectiveness of the PMT program, these results enhance the integrity of follow-up analyses conducted with the intervention group only.

**Figure 2.**
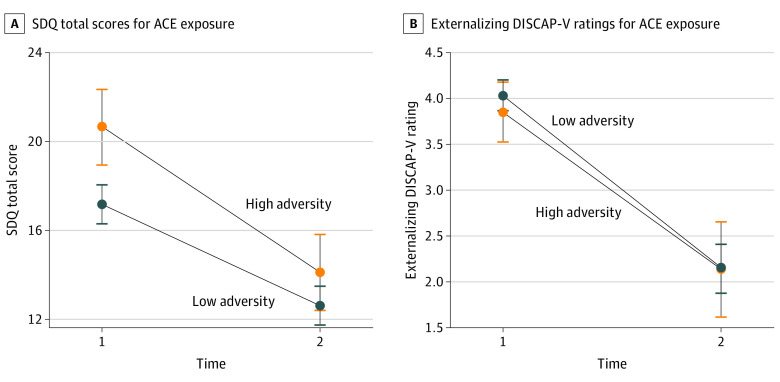
Treatment Outcomes by Adverse Childhood Experience (ACE) Exposure Plots comparing high and low ACE exposure Strengths and Difficulties Questionnaire (SDQ) and externalizing Diagnostic Interview Schedule for Children, Adolescents, and Parents, 5th Edition (DISCAP-V) mean ratings at time 1 and time 2 for outcomes with significant time × ACE interactions for SDQ. Error bars indicate 95% CIs.

**Figure 3.**
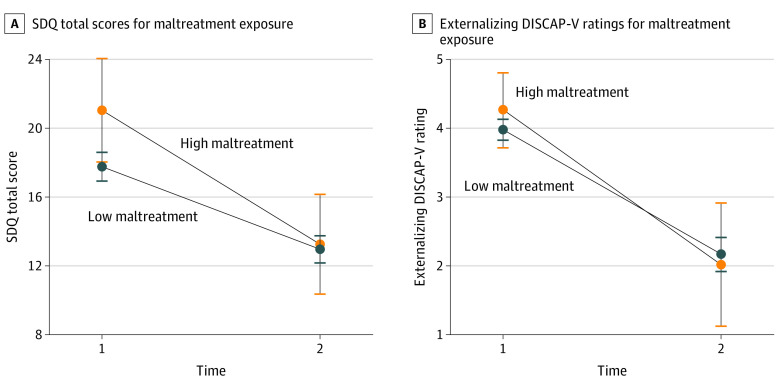
Treatment Outcomes by High vs Low Exposure to Maltreatment Plots comparing high and low exposure to maltreatment Strengths and Difficulties Questionnaire (SDQ) and externalizing Diagnostic Interview Schedule for Children, Adolescents, and Parents, 5th Edition (DISCAP-V) mean ratings at time 1 and time 2 for outcomes with significant time × group interactions for SDQ. Error bars indicate 95% CIs.

Within the intervention group, children were stratified into high and low ACE exposure, and maltreatment conditions and outcomes were compared between groups. The significant interaction effect of time and adversity *(F*_1,149_ = 4.45; *P* < .04) revealed that children with high ACE exposure had higher baseline SDQ scores compared with children with low ACE exposure (mean difference, 3.46 [95% CI, 1.51-5.41]; *P* < .001); however, posttreatment outcomes were equivalent across both groups (mean difference, 1.49 [95% CI, −0.46 to 3.44]; *P* = .13) (Figure 2; eTable 2 in Supplement 2). Similarly, a significant interaction effect (*F*_1_*_,_*_141_ = 3.97; *P* < .05) found that children with high exposure to maltreatment had higher baseline SDQ scores compared with children with low exposure to maltreatment (mean difference, 3.28 [95% CI, 0.14-6.42]; *P* = .04) and that there were equivalent posttreatment SDQ outcome scores between groups (mean difference, 0.29 [95% CI, −2.72 to 3.30]; *P* = .85) (Figure 3; eTable 3 in Supplement 2). No between-participant differences in externalizing DISCAP-V ratings were found.

**Figure 4.**
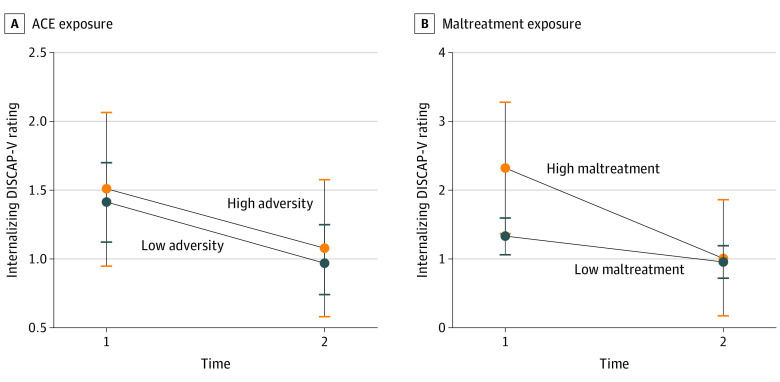
Internalizing Diagnostic Interview Schedule for Children, Adolescents, and Parents, 5th Edition (DISCAP-V) Ratings by Exposure to Adverse Childhood Experiences (ACEs) and Exposure to Maltreatment Plots comparing high and low ACE exposure internalizing symptom outcomes with significant time × group interactions for internalizing DISCAP-V severity. Error bars indicate 95% CIs.

Last, a significant interaction effect (*F*_1,141_ = 3.92; *P* = .05) revealed that the high maltreatment exposure group had higher baseline internalizing DISCAP-V ratings compared with children in the low maltreatment exposure group (mean difference, 1.00 [95% CI, −2.00 to 0.00]; *P* = .05); however, both groups showed similar posttreatment internalizing DISCAP-V outcomes (mean difference, 0.06 [95% CI, −0.82 to 0.94]; *P* = .90) (Figure 4; eTable 3 in Supplement 2).

## Discussion

This study found that, compared with children with low ACE exposure, children with high ACE exposure experience equivalent, if not greater, therapeutic benefit associated with PMT-TO programs. Contrary to the hypotheses, children with high ACE exposure and children with low ACE exposure experienced equivalent reductions in externalizing symptoms, decreasing from the clinical range at baseline to the nonclinical range after treatment. The study findings extend prior research demonstrating the equivalent efficacy of PMT-TO programs with the child-welfare community^41,42^ to show that children with high adversity exposure displayed greater reductions in CMH problems and internalizing symptoms compared with peers with low adversity exposure.

The results contribute to the debate surrounding PMT-TO programs among the population of children with ACEs, with central concerns pertaining to the potential for TO to exacerbate emotion regulation deficits and attachment ruptures.^24^ Biopsychosocial models of child development suggest that aggravating such deficits would result in an increase in CMH symptoms.^39,40^ Our findings suggest, however, that not only do PMT-TO programs appear to not exacerbate CMH problems among the population of children with ACEs, but they may be especially beneficial for these children, who often present with more severe symptoms prior to intervention.^41^

There are several possible mechanisms associated with these outcomes. Because the evidence-based application of TO is embedded within a suite of other parenting techniques, it is possible that the benefits associated with PMT-TO programs for the high ACE groups were due solely to program components outside of TO and that these components may have even overridden the potential negative outcomes of TO. However, this potential is unlikely given the meta-analytic findings that TO is a core active ingredient associated with the effectiveness of PMT.^26,58^ It is also possible that the reductions in CMH symptoms were fear induced, being more pronounced among children in the high ACE group owing to heightened perceptual biases to hostility and threat.^1^ If fear of TO was the factor associated with change, one would expect to see an increase in fear-related symptoms^59^; however, the opposite was found when children with high maltreatment exposure experienced reductions in internalizing symptoms.

An alternative trauma-informed explanation is that the PMT-TO programs may be particularly reparative for children exposed to ACEs. In high-risk parent-child dyads, adversity often occurs in the context of violent or dysfunctional discipline.^60^ Although the effectiveness of TO rests on the quality of parent-child interactions outside of discipline, evidence-based implementation of TO shifts the home climate from one of unpredictability, reactivity, and hostility to one where controlled, consistent, and reasonable emotional responses are modeled. Children exposed to more severe adversity may exhibit more pronounced responsiveness to a PMT-TO program as they observe and internalize caregivers’ emotion regulation skills^1^ and “replace the distress and fear that was once associated with discipline with feelings of safety, security, and predictability.”^24^^(p11)^

The ability to effectively repair is said to be the hallmark of secure attachments.^61^ In addition to other core attachment-building PMT strategies, TO may be particularly relevant to healing attachments for children with high ACE exposure because TO provides families with a structured plan for facilitating attachment repairs, instead of causing further damage through harsh discipline or “abandoning” their child by shutting down or stonewalling.^31^ Parents do not abandon their child during TO because the effective implementation of TO requires parents to be inextricably present during the procedure, intervening, monitoring, timing, releasing, and repairing throughout. Although TO requires the removal of caregiver attention for a short time, when this process is done effectively, it mirrors a secure attachment, being a successful separation and reunion without threatening attachment bonds.^62^

The results of this study support the use of a PMT-TO program for children with ACE exposure, which is reassuring because TO is a core component of the most widely implemented intervention for children with ACE exposure, trauma focused–cognitive behavioral therapy,^63,64^ as well as many attachment-based programs designed specifically for the child-welfare population.^65^ These results do not capture the outcome of TO in the community, where it can be misused. There is always potential for TO to cause harm if it is implemented inconsistently, harshly, and/or in the absence of ample positive parent-child interactions.

### Limitations

The study has several limitations. Because it was geographically limited to Sydney, Australia, these results cannot be generalized to other populations nationally or internationally. The sample was nonrandonmized to intervention vs waiting list conditions, introducing bias to interpretations of group difference. Bias may also have occurred in caregiver reports of child adversity, where caregivers may have declined reporting certain ACEs for fear of negative consequences, weakening the observed associations. Conversely, because caregivers provided data on both ACE exposure and outcomes, common method bias may have inflated the association between these variables.^66^ The small group with high exposure to maltreatment reduced the power of these analyses and increased the margin of a type II error.^57^ Although it would be unethical and inaccurate to evaluate TO as a standalone strategy, future research could assess the individual association of TO with attachment and emotion regulation with greater precision through the inclusion of specific measures of these constructs and observational data.

## Conclusions

This nonrandomized clinical study compared the benefits and potential harms associated with PMT-TO programs for children exposed to adversity and found that children with high ACE exposure experienced equivalent, if not greater, reductions in behavioral and emotional difficulties than children with low ACE exposure. This study has prompted further investigation to address the controversy surrounding TO.

## Notes

Supplement 1.
Study Protocol

Supplement 2. **eFigure.** CONSORT Flow Chart of the Study **eTable 1.** Repeated Measures ANOVA - Time x Condition, Time Period (T), Means (M), Standard Deviations (SD), Mean Differences Between Groups (MD), 95% Confidence Intervals (CI), and P Value (P) **eTable 2.** Repeated Measures ANOVA - Time x ACE Exposure, Time Period (T), Means (M), Standard Deviations (SD), Mean Differences Between Groups (MD), 95% Confidence Intervals (CI), and P Value (P) **eTable 3.** Repeated Measures ANOVA - Time x Maltreatment (MI) Exposure, Time Period (T), Means (M), Standard Deviations (SD), Mean Differences Between Groups (MD), 95% Confidence Intervals (CI), and P Value (P)

Supplement 3.
Data Sharing Statement
