# Supplementary material for: Using Time-out for Child Conduct Problems in the Context of Trauma and Adversity: A Nonrandomized Controlled Trial
Source: JAMA Netw Open. 2022 Sep 1;5(9):e2229726. doi: 10.1001/jamanetworkopen.2022.29726 (PMC9437765; doi:10.1001/jamanetworkopen.2022.29726)
Supplement: Supplement 1. — Study Protocol [file jamanetwopen-e2229726-s001.pdf]

Sydney Child Behaviour Research Clinic

STUDY PROTOCOL (Internal use)

The REAL Treatment Study: A Parenting Intervention for Child Conduct Problems

CONTENTS

The Research Team……………………………………………………………………..3

The Project Rationale…………………………………………………………………...4

Research Questions and Aims…………………………………………………………..4

Research Protocol Summary……………………………………………………………5

Recruitment and Screening……………………………………………………..6

Participant Information and Consent…………………………………………...6

Pre-Treatment Assessment……………………………………………………...7

Feedback and Treatment………………………………………………………..9

Post-Treatment Assessment……………………………………………………9

Follow-Up Assessment…………………………………………………………...11

Study Site Details………………………………………………………………………11

Data Collection and Storage…………………………………………………………...11

Data Analysis…………………………………………………………………………..12

Study Monitoring, Safety and Adverse Events………………………………………..13

Potential Significance of the Study……………………………………………………14

References……………………………………………………………………………..15

Appendices……………………………………………………………………………16+

THE RESEARCH TEAM

Internal Chief Investigators

Professor Mark Dadds
Project Director and Chief Investigator
mark.dadds@sydney.edu.au

Dr David Hawes
Project Director and Chief Investigator
david.hawes@sydney.edu.au

Project Manager and Investigator

Dr Meryn Lechowicz
Project Manager and Senior Psychologist
meryn.lechowicz@sydney.edu.au

Project Research Assistant and Clinician

Alex Roach
Clinical Psychology Registrar/Research Assistant
alex.roach@sydney.edu.au

External Chief Investigators

Dr Divya Mehta
Chief Investigator
divya.mehta@qut.edu.au

Dr Eva Kimonis
Chief Investigator
e.kimonis@unsw.edu.au

Professor Paul Frick
Chief Investigator
pfrick@lsu.edu

Postgraduate Student Investigators:

Jaimie Northam
Psychology PhD Candidate
jnor0234@uni.sydney.edu.au

Carri Ann Fisher
Psychology PhD Candidate
cfis7323@uni.sydney.edu.au

PROJECT RATIONALE

The origins of many mental health disorders are identifiable early in life, and early intervention programs are effective in significantly reducing lifetime impairments. This is a major achievement of the health sciences, but is offset by evidence that the most effective of these interventions (parent training for conduct problems, and CBT for anxiety disorders) only produce clinically significant change in ~50% of cases. Outcomes are considerably worse for children with multiple comorbid problems, particularly the neurodevelopmental disorders of ADHD and Autism Spectrum Disorders (ASD). Research from our clinic replicated in international samples has shown that individual differences in the epigenetic regulation of specific neurodevelopmental genes map onto and predict individual differences in social-Responsiveness, Emotional Attention, and Learning (REAL), which in turn, precisely characterise emerging psychiatric symptom patterns in children with complex mental health problems. Importantly, there are good reasons to expect that the interplay of these systems will predict the child's responsiveness to evidence-based intervention, and in turn, be altered by early intervention.

In order to improve treatments for child psychopathology, it is necessary to understand the neurodevelopmental processes that underpin clinical change during the periods in which the earliest features of mental health problems are most responsive to intervention. This study proposes to utilize progress in epigenetics and neurodevelopment to conduct a prospective longitudinal cohort study that will inform the treatment needs of individual children with complex presentations.

RESEARCH QUESTIONS AND AIMS

This study has been designed to test the interplay of responsiveness to intervention, genetics and neurodevelopment to: a) confirm an emerging model of how epigenetic regulation of the major neurodevelopmental systems maps onto individual differences in comorbid symptom profiles before and after intervention; b) to examine how this regulation and mapping predicts individual differences in responsiveness of children to intervention that produces standardised and measurable improvements in the child caregiving environment; c) to examine how early intervention alters epigenetic regulation of the major neurodevelopmental systems and REAL phenomena, thus modifying risk versus protection for future development.

RESEARCH PROTOCOL:

The research project will be a single site study and will take place at the Sydney Child Behaviour Research Clinic (SCBRC). Potential participants are the parents seeking help for managing behaviour problems in their child(ren).

Recruitment and Screening

Initial contact (**intake form** and telephone information)
Extended screening assessment (for lengthy wait times):

1. Clinical Interview and the Diagnostic Interview Schedule for Children, Adolescents, and Parents (DISCAP) Assessment
2. Strengths and Difficulties Questionnaire (SDQ) (Goodman, 1997)
3. Amended Primary Attachment Style Questionnaire (PASQ; Salzman, Kunzendorf, Saunders, & Hulihan, 2013)
4. Methylation Assessment: Buccal Cell Collection

Participant Information and Consent

1. Parent/Guardian
2. Teacher (sent with questionnaire link)

Pre-Treatment Assessment

1. Clinical Interview and the Diagnostic Interview Schedule for Children, Adolescents, and Parents (DISCAP) Assessment
2. Online (or paper) Questionnaires
    a. Parent/Caregiver Questionnaires
    b. Teacher/Day care staff Questionnaires
    c. Clinician Questionnaires
3. Computer Assessment: FACES Task
4. Family Observation Tasks and Psychophysiological Measures
5. Methylation Assessment: Buccal Cell Collection
6. Cognitive Assessment: The Peabody Picture Vocabulary Task (PPVT)

Treatment

Parent Management Training: **Mark R. Dadds and David J. Hawes:** INTEGRATED FAMILY INTERVENTION FOR CHILD CONDUCT PROBLEMS: A Behaviour-Attachment-Systems Intervention for Parents

Mid-Point Methylation Assessment

Post-Treatment Assessment

1. Brief Clinical Interview & DISCAP Assessment (Blind assessor)
2. Online Questionnaires
    a. Parent/Caregiver Questionnaires
    b. Teacher/Day care staff Questionnaires

    c. Clinician Questionnaires (dose and engagement measures)
3. Computer Assessment: FACES Task
4. Family Observation Tasks and Psychophysiological Measures
5. Methylation Assessment

Follow-Up Assessment
Brief Clinical Interview and DISCAP Assessment (Blind assessor)

Recruitment and Screening:

Initial Contact:

Families will be identified for recruitment and intake from our regular pool of self-referred families to the SCBRC. Typically parents have heard of the CBRC via schools, their GP or other health practitioner, or have found us during searches online or have responded to occasional notices we place in local magazines such as Sydney's Child that provide brief factual information about the research project. At first contact the parents are involved in a full 'arms length' information giving session involving details of the CBRC processes as well contact details for alternative non-research treatments should the parents prefer, and a screening assessment as to their suitability according to inclusion and exclusion criteria specified below, this takes approximately 15 minutes to complete. From this screening interview, likely eligible families are then allocated as potential participants for the research project and invited to attend an initial appointment to review information about the research project and consent documents. Those who wish to proceed and give full informed consent will undergo a comprehensive assessment. Extended screening assessments are offered to families where lengthy wait times prior to commencing treatment are expected. This is to ensure that ineligible families are not forgoing other treatment options while having to endure a lengthy wait for the initial assessment. The 1-hour face-to-face screening interview involves a semi-structured interview (Diagnostic Interview Schedule for Children, Adolescents, and Parents [Holland & Dadds, 1997]), a short behavioural screening questionnaire (the Strengths and Difficulties Questionnaire [Goodman, 1997]) and the collection of a buccal cell sample.

Inclusion Criteria:

Children aged between 2 to 8 years (no gender-based exclusion) who are assessed as having conduct problems using the following criteria: primary DSM-V diagnosis of Oppositional-Defiant or Conduct Disorder.

We adhere to the NIMH guidelines that research into the externalising disorders is compromised by exclusion of the major and naturally occurring comorbidities, thus ADHD, affective/anxiety disorders, learning problems, and mild autistic features are allowed if they are secondary and less severe than the conduct problems, and the child is not receiving or is stabilised on psychoactive medication in relation to them at the time of assessment.

Exclusion Criteria:

Exclusionary criteria include presence of a psychotic disorder, intellectual disability resulting in IQ 70, or a major medical disorder that has significantly interfered with school/family life, or family involvement in engaged in current legal issues including child custody disputes. Fluency in English will also be a criterion for inclusion due to the language format of the psychological measures.

Participant Information and Consent Forms

Consent for the family to participate in the research will be obtained from the parents/guardians of the referred children. As described above families have been made aware of the CBRC as a research clinic prior to attending an initial appointment. At the initial appointment the family are then provided with further information about the research in the form of participant information forms as approved by HREC. Families are given time to review this information, seek clarification or discuss any questions or concerns with a member of the CBRC and also offered the opportunity to contact the project manager or chief investigator should that have any further questions prior to signing consent forms. All members of the CBRC who obtain consent from families, are given appropriate training in informed consent procedures by the project manager. Families also complete Consent to consult form prior to any member of the CBRC contacting a participant’s school or daycare center as part of the research.

Teacher/child care worker consent to participate is sought using a written participant information form and consent form as approved by HREC which is sent to the teacher/child care worker once consent to contact this professional has been provided by the parent/caregiver.

Pre-Treatment Assessment

- **Clinical Interview and Diagnostic Assessment:**

Parents/caregivers are invited to attend the first appointment at the CBRC without their child/ren and a clinical interview is conducted. Basic demographic and background details are also gathered during this appointment including child date of birth, age, gender, medication status, ethnicity of biological paternal and maternal grandparents (when known as required for epigenetic analysis) languages spoken at home, parent/caregiver age, gender, relationship to child, level of education, employment status and relationship between caregivers. This appointment also involves completion of appropriate sections of the Diagnostic Interview Schedule for Children, Adolescents, and Parents for DSM V (Avalon, Lechowicz, Hawes & Dadds, 2017), a measure of diagnostic status of the child. Parents/caregivers are then given either paper copies or online links to standardized questionnaires to complete.

- **Online/Paper Copy Questionnaires:**

   a. ***Parent/Caregiver Questionnaires***

- ADHD Rating Scales (DuPaul, 1991; McGoey, DuPaul, Haley & Shelton, 2007)
- Strengths and Difficulties Questionnaire (SDQ) (Goodman, 1997)
- Antisocial Process Screening Device (ASPD) (Frick & Hare, 2001)
-

- Spence Child Anxiety Scale Parent Report and Pre-School versions (SCAS) (Nauta, Scholing, Rapee, Abbott, Spence & Waters, 2004; Spence & Rapee, 1999)
- The Autism Spectrum Quotient 10 Child Version (AQ10 Child) (Auyeung & Baron-Cohen, 2012)
- Parenting and Family Adjustment Scales (PAFAS) (Sanders, Morawska, Haslam, Filus & Fletcher, 2013)
- Parent Problem Checklist (Dadds & Powell, 1991)
- Parent Feelings Questionnaire (Deater-Deckard, 1996)
- The Depression, Anxiety and Stress Scales 21 Item Version (DASS 21) (Lovibond & Lovibond, 1995a)
- The Adapted Adverse Experiences Scales, Self and Caregiver Report Versions (Lechowicz, Roach, Fisher, Hawes & Dadds, 2017)
- Oppositional Defiant Disorder Irritability and Hurtfulness Dimensionality Measure
- Amended Primary Attachment Style Questionnaire (PASQ; Salzman, Kunzendorf, Saunders, & Hulihan, 2013)
- Evidence-based Time-out Implementation Scale (ETIS)
- Child Stress Disorders Checklist- Short Form (CSDC-SF; Enlow, Kassam-Adams & Saxe, 2010)

b. Teacher/Child Care Worker Questionnaires

- ADHD Rating Scales (DuPaul, 1991; McGoey, DuPaul, Haley & Shelton, 2007)
- Strengths and Difficulties Questionnaire (SDQ) (Goodman, 1997)
- Antisocial Process Screening Device (ASPD) (Frick & Hare, 2001)

c. Clinician Questionnaires

- Maltreatment Index (based on the Maltreatment Classification System by Barnett et al., 1993; 3 items rating levels of emotional and physical abuse and neglect)
- The Quality of Family Environment **Quality of the Family Environment** – a rating given by the clinician using a global scale to measure the quality of the family environment (Rey et al., 1997)

- **Computer Assessment: FACES Task**

UNSW FACES protocol (Facial Emotion Task) (Dadds et al., 2008, 2011) is a PowerPoint presentation in which participants identify the emotion of the face from the six target emotions of sadness, happiness, neutral, anger, fear and disgust, using 36 faces (6 actors x 6 emotions). The task has undergone extensive validation using child and adult samples. Dependent measures are eye gaze to the eye region of the stimuli faces, galvanic skin conductance and heart rate, and overall accuracy for each emotion under each gaze condition. Psychophysiological measures of the referred child including pupil dilation, eye gaze, galvanic skin conductance and heart rate are gathered during this task.

- **Family Observation Tasks and Psychophysiological Measures**

Structured observations of family interactions will be used to index emotion-related parent-child behaviours that are not possible for parents to accurately self-report on. Observational activities will comprise structured age-appropriate play interactions and a brief (e.g., 10-minute) emotion-reminiscing task (‘‘talk about a happy time that you have all shared together and a sad time that you have all shared together”; Fivush, Marin, McWilliams, & Bohanek, 2009). Families will also view a small exert of the Lion King film, which is commonly used to elicit responses to emotional scenes. These activities are video recorded and coded. Psychophysiological measures of the referred child including pupil dilation, eye gaze, galvanic skin conductance and heart rate are gathered during the emotion elicitation exercise.

Family observation tasks include:

a. Parent-child free play warm up exercise
b. Emotion elicitation exercise (Control and Lion King videos)
c. Parent-child emotion processing task
d. Child only comprehension tasks
e. Eye-love you task
f. Parent Only Speech Sample Observation Tasks

- **Methylation Assessment: Buccal Cell Collection**

Buccal cell samples will be gathered from assenting children in order to measure functioning, via genetic sequencing, epigenetic markers, and peripheral levels, on the major neurohormonal systems of oxytocin/vasopressin, serotonin, dopamine, cortisol, and testosterone. Oracollect DNA OCR-100 swab kits will be used to collect Buccal cell samples however the children can refuse to participate in this. Participants are fully informed that these samples are only used for the neurohormomal assays and provide no information about paternity, health conditions, or other controversial information.

The samples will be collected by trained researchers and clinicians in the Child Behaviour Research Clinic using Oracollect DNA OCR-100 swab kits. However if children refuse to have a trained researcher or clinician collect the sample they will be given the option for parents to collect the sample should this be preferred. In such circumstances parents will be briefed as to the protocol for collecting Buccal cell using the swabs as employed by the researchers and clinicians.

- **Cognitive Assessment: The Peabody Picture Vocabulary Task (PPVT) (Dunn & Dunn, 2007)**

A developmental screening tool to estimate global abilities selected to be appropriate for the child’s age from Peabody Picture Vocabulary Test (PPVT) (Dunn & Dunn, 2007).

Feedback and Treatment

Parents/caregivers are then invited back for a feedback and treatment planning session in which clinicians provide feedback on the assessment process and engage parents in a partnership empowering parents to engage with assessment findings and take part in intervention planning. Eligible families are then offered treatment. All families will be offered 'treatment as usual' there will be no control condition, no waitlist condition and no random allocation of cases within the proposed research project. The treatment comprises the Dadds and Hawes (2006) program, an evidence-based intervention with a long empirical history in the treatment of early-onset behavioural and emotional difficulties in children.

Treatment consists of approximately 6 to 10 x 1 to 1.5 hour treatment sessions that focus on empowering parents to better manage child behavioural difficulties, as well selected modules on parental mental health, marital adjustment and family communication and additional modules to address specific concerns such as management of anxiety and worry and emotional development or emotion socialization and school consultation where indicated for children and families.

Mid-point Methylation Assessment: Buccal Cell Collection (procedure as described above)

Post-Treatment Assessment

Post-treatment assessments are completed on average approximately 3 months after pre-treatment measures have been collected. This will involve completing the following assessments:

1. **Diagnostic Assessment**

The Diagnostic Interview Schedule for Children, Adolescents, and Parents (Holland & Dadds, 1997) is completed by a clinician who is blind to the pre-treatment diagnostic status of the child referred.

2. **Online/Paper Copy Questionnaires:**

   a. ***Parent/Caregiver Questionnaires***

- ADHD Rating Scales (DuPaul, 1991; McGoey, DuPaul, Haley & Shelton, 2007)
- Strengths and Difficulties Questionnaire (SDQ) (Goodman, 1997)
- Antisocial Process Screening Device (ASPD) (Frick & Hare, 2001)
- Spence Child Anxiety Scale Parent Report and Pre-School versions (SCAS) (Nauta, Scholing, Rapee, Abbott, Spence & Waters, 2004; Spence & Rapee, 1999)
- The Autism Spectrum Quotient 10 Child Version (AQ10 Child) (Auyeung & Baron-Cohen, 2012)
- Parenting and Family Adjustment Scales (PAFAS) (Sanders, Morawska, Haslam, Filus & Fletcher, 2013)
- Parent Problem Checklist (Dadds & Powell, 1991)

- Parent Feelings Questionnaire (Deater-Deckard, 1996)
- The Depression, Anxiety and Stress Scales 21 Item Version (DASS 21) (Lovibond & Lovibond, 1995a)
- The Adapted Adverse Experiences Scales, Self and Caregiver Report Versions
- Parent/Caregiver treatment engagement questionnaire (used in the Child Behaviour Research Clinic)
- Amended Primary Attachment Style Questionnaire (PASQ; Salzman, Kunzendorf, Saunders, & Hulihan, 2013)
- Evidence-based Time-out Implementation Scale (ETIS)
- Child Stress Disorders Checklist- Short Form (CSDC-SF; Enlow, Kassam-Adams & Saxe, 2010)
- Parent perceptions of Oppositional Defiant Disorder (PPODD)

b. Teacher/Child Care Worker Questionnaires

- ADHD Rating Scales (DuPaul, 1991; McGoey, DuPaul, Haley & Shelton, 2007)
- Strengths and Difficulties Questionnaire (SDQ) (Goodman, 1997)
- Antisocial Process Screening Device (ASPD) (Frick & Hare, 2001)

c. Clinician Questionnaires

- Maltreatment Index (based on the Maltreatment Classification System by Barnett et al., 1993; 3 items rating levels of emotional and physical abuse and neglect)
- The Quality of Family Environment **Quality of the Family Environment** – a rating given by the clinician using a global scale to measure the quality of the family environment (Rey et al., 1997)
- Clinician characteristics (e.g. years of experience) (used in the Child Behaviour Research Clinic)
- Treatment Fidelity Checklist (used in the Child Behaviour Research Clinic)
- Clinician rated engagement measure

3. **Computer Assessment:** FACES Task as described above.

4. **Family Observation Tasks and Psychophysiological Measures:**

   a. Parent-child free play warm up exercise
   b. Parent Only Speech Sample Observation Tasks

5. **Methylation Assessment:** Buccal Cell Collection as described above.

<u>Follow-Up Assessment:</u>

A brief follow-up assessment is then conducted a further 3 months after post-treatment assessments have been conducted. This follow-up assessment takes the form of a face to face assessment in which a trained clinician who is blind to the referred child's pre-treatment and post-treatment

diagnostic status conducts a The Diagnostic Interview Schedule for Children, Adolescents, and Parents (Holland & Dadds, 1997) and asks for any verbal feedback from the family. At this time families requesting further advice or support will be offered information about appropriate services to meet their needs.

STUDY SITE DETAILS:

Clinical participants will be clients of the Sydney Child Behaviour Research Clinic at the University of Sydney. The Street address for the SCBRC is Building M02K, 97 Church Street, Brain and Mind Centre, Camperdown NSW 2050.

DATA COLLECTION AND STORAGE:

Data collection and storage will be overseen by the project manager for the duration of the study. Data obtained from families over the course of assessment, intervention and follow-up that pertain to the clinical procedures involving patient care will be recorded in the patient file and relevant information will be extracted and stored in a data base as part of the study deidentified data set.

Information provided by study participants in the form of parent completed measures will be obtained using online and or paper surveys. Information from online surveys will be extracted from the online survey host site (e.g. REDCap) into the data set and a data summary form of all clinically relevant information obtained from these measures will be entered into the patient clinical file. The patient files will form the source files against which data set information can be checked.

All data will be safely stored under secure storage. Non-digital Data (including participant clinical files) will be stored inside a locked cabinet in a locked room inside the Sydney Child Behaviour Research Clinic in the Storage/Filing Room 127 in building M02K, at 97 Church Street, Brain and Mind Centre, Camperdown NSW 2050. Online data will be stored in secure university-based data storage server system in the same file storage room in the SCBRC. Access to these documents is restricted to employees of the clinic who are all briefed in the handling of sensitive information. Online data will be stored in secure servers in the same file storage room in the SCBRC.

Buccal cell samples will be de-identified and stored in the Sydney Child Behaviour Research Clinic File StorageRoom 127 in building M02K, at 97 Church Street, Brain and Mind Centre, Camperdown NSW 2050. This room will be locked at all times, keys will be held by the two PIs. When samples are sent for analysis they will be sent to the Australian Genome Research Facility Ltd using a university approved courier service that requires the receiver to sign for the receipt of the package on its arrival. All samples will be destroyed at the completion of the research. All digital data pertaining to the samples will be stored in secure university-based data storage server system in the same file storage room in the SCBRC.

DATA ANALYSIS:

<u>General Analysis Plan:</u>

The study is a prospective longitudinal cohort study and will take place from the point of approval and trial registration (likely to be approximately July 2017). The main science and analyses centre around using diagnostic grouping (Disruptive Behaviour Disorders, Anxiety and Depression, ADHD, Autism Spectrum Disorder) as the predictors and genetic and epigenetic data, neurohormonal levels, patterns of emotion processing, family interactions, cognitive impairments, and health outcomes being used as criterion variables. Typically we use Generalised Estimating Equations or similar multivariate methods with diagnostic groupings modelled as both categorical and dimensional variables in order to address the science of formal psychiatric categorical diagnoses, but also tackle the real world issue that diagnostics are rarely discrete and in fact, show high overlap and comorbidities and should be modelled as continuous and overlapping variables.

With respect to specific statistical design for data collection and analysis, the core design yields a set of repeated measures of the REAL constructs, overall methylation levels for up to 14 genes, indices of parent/family function and child psychopathology, and treatment dose. Missing data will be examined for non-random patterns and an appropriate missing data strategy used to model any trends found. Random missing data will be managed using maximum likelihood estimation within the mediational modeling. The main regression analyses will be conducted using the contextual variables of environmental adversity, and non-child predictors of treatment dose and outcomes as covariates. The mediation model will be tested according to the model described by Preacher and Kelly for longitudinal multivariate mediational designs (Preacher & Kelley, 2011). Our parameters for input were calculated based on known effect sizes from the studies describing methylation change associated with psychotherapy described above (moderate effect sizes of regression weights 0.2-0.4), our own pilot data on individual differences in methylation levels for oxytocin, DRD4, and cortisol receptors (expect a 15% change in methylation from clinical to non-clinical status, estimated effect size 0.3), and changes in REAL phenomena over the course of our intervention (moderate to large effect size of 0.5 in improvement in emotional attention, responsiveness and learning (Dadds et al., 2012). The sample sizes needed to detect full, partial mediation (50%), and accept zero mediation are N = 225, 245, and 320 according to the Preacher and Kelly formulae. Our study is designed with greater power than these because we also have to factor in various control variables such as age, ethnicity, non-child variables that predict treatment engagement such as family chaos, and random employment, illness, vacation interference with treatment.

<u>Methylation Analysis:</u>

Indexation of methylations levels will be conducted on buccal cell DNA, considered the most informative index of both neural and system-wide states analysed by the AGRF using the Infinium Human Methylation EPIC850K BeadChip Kit. Buccal cell samples will be gathered from assenting children in order to measure functioning, via genetic sequencing, epigenetic markers, and peripheral levels, on the major neurohormonal systems of oxytocin/vasopressin, serotonin, dopamine, cortisol, and testosterone.

The final methylation data produced represents the percentage methylation at each single-nucleotide CpG site tested, representing thousands of data points, and will be managed using three complementary approaches. First, the study will test specific hypotheses about methylation of individual CpG sites chosen a priory on the basis of existing findings specifying their associations

with functional neural transcription as well as broader phenotypes in psychopathology. Second, and for the purposes of the main mediation analyses, we will compute indices of 'whole gene' methylation levels.

Importantly, while the study is not focused on sequence variations in the target genes, these data are needed to control for variations that affect methylation assays: CpG targets that are single nucleotide polymorphisms (SNPs) and vary by ethnicity, this is also why we collect demographic data concerning the biological grandparents ethnicity of the referred child. Participants are fully informed that these samples are only used for the neurohormomal assays and provide no information about paternity, health conditions, or other controversial information.

Family Observation Tasks and Psychophysiological Measures:

Positive and negative parent-child behaviours and emotional responses in family observation activities will be coded based on coding systems previously developed by our team (Dadds, Gale, Godbee, Moul, Pasalich, Fink, & Hawes, 2016; Pasalich, Waschbusch, Dadds, & Hawes, 2014; Hawes et al., 2013). Key aspects of the parent-child relationship will also be coded from a brief (e.g., 10 minute) speech-sample recording of parents describing their child in the absence of the child. This will be coded using the Family Affective Attitude Rating Scale (see Weston, Hawes, & Pasalich, 2016), and the Mind-Mindedness Coding Manual to assess parents mental, behavioural, physical, and general attributions about their child (Meins & Fernyhough, 2015).

STUDY MONITORING, SAFETY AND ADVERSE EVENTS:

Study monitoring to ensure patient safety and data integrity will be overseen by the project manager and where appropriate the chief investigators. The project manager will ensure that all consent forms have been signed and dated appropriately and will review a random sample of data in source files and check against the data set. In instances where the project manager is involved in the provision of clinical services to a trial participant, the monitoring of these files will be overseen by either the study research assistant or one of the investigators on the trial.

The project manager and CI's will also monitor and oversee the management and reporting of any risk concerns raised during the course of any activities associated with the research project and ensure that all clinic members are informed of procedures for addressing and reporting risk concerns. There are no expected adverse events associated with the study. The study does not involve a drug or device but is a study involving a psychotherapeutic/ behavioural therapy. Any unanticipated adverse events will be documented and the HREC and Clinical Trials Governance committee will be notified of these.

Where a risk issue involves risk of significant harm to a child or young person, as per our professional mandatory reporting responsibilities the appropriate bodies (Family and Community Services, Child Protection Services or NSW Police) will also be notified of this event or concerns. All adverse events will also be documented in the Trial log, and where appropriate in a recruited family's clinical file. The research is not designed to uncover such activities but does have the potential to uncover suspected child abuse and neglect by parents. This is unlikely as parents have actively and voluntarily approached the SCBRC for assessment and help, however it is possible.

Ultimately, the discovery of such suspected activity is a good thing for the parents and child involved. Any information pertaining to the risk or safety of a child will be reported to the project manager or clinic directors who as registered clinical psychologists are trained to manage the discovery of such information in a clinically ethical and sensitive manner, and are legally mandated to report to relevant state authorities. Such information is not kept confidential. As mental health professionals, we are legally mandated to report suspected child abuse and neglect.

This is a psychological research study. All participants will be directed to contact emergency services in the event of a medical emergency or their local GPs in the event of a medical concern that is not an emergency. Should any study participant experience a medical issue or emergency at the research site standard university wide workplace health and safety procedures apply and immediate medical attention or care will be sought for the individual where necessary and appropriate.

POTENTIAL SIGNIFICANCE OF THE STUDY:

This project will map stability and change in individual emotional and behavioural factors and associated epigenetic status in a representative sample of children referred for common mental health problems as they progress through an evidence-based intervention that maximises the quality of the child-rearing environment. The study will be the first to test a clear developmental map of both the unique and specific causes of psychopathology and will identify more precise early intervention targets for children with complex comorbid conditions. The research has the potential to improve theoretical models, assessment strategies, and treatment approaches, and thus, positive outcomes for children with early-onset behavioural and mental health problems.

REFERENCE:

Barnett, D., Manly, J. T., Cicchetti, D. (1993). Defining child maltreatment: The interface between policy and research. In: Cicchetti D, Toth SL, editors. Child abuse, child development, and social policy. Ablex; Norwood, NJ, 7–73.

Brestan, E. V., & Eyberg, S. M. (1998). Effective psychosocial treatments of conduct-disordered children and adolescents: 29 years, 82 studies, and 5,272 kids. Journal of clinical child psychology, 27(2), 180-189.

Squires J, Bricker D. (2009). Ages & Stages Questionnaires Third Edition (ASQ- 3 TM). A parent-completed child-monitoring system. Baltimore: Paul H. Brookes Publishing Co.

Conners, C. K. (1997). The Conners Rating Scales – Revised manual. North Towanda, NY: Multi-health Systems.

Dadds, M. R., Gale, N., Godbee, M., Moul, C., Pasalich, D., Fink, E., Hawes, D.J. (2016). Expression and regulation of attachment-related emotions in children with conduct problems and callous-unemotional traits. Child Psychiatry & Human Development, 47(4), 647-656.

Dadds, M. R., & Hawes, D. J. (2006). Integrative Family Intervention for Child Conduct Disorders.

Dadds, M. R., Hunter, K., Hawes, D. J., Frost, A. D., Vassallo, S., Bunn, P., ... & El Masry, Y. (2008). A measure of cognitive and affective empathy in children using parent ratings. Child psychiatry and human development, 39(2), 111-122.

Dadds, M. R., Jambrak, J., Paslich, D., Hawes, D., Brennan, J. (2011). Impaired attention to the eyes of attachment figures and he developmental origins of psychopathology.. Journal of Child Psychology and Psychiatry. Vol.52(3), 238-245.

Dadds, M. R., & Powell, M. B. (1991). The relationship of interparental conflict and global marital adjustment to aggression, anxiety, and immaturity in aggressive nonclinic children. Journal of Abnormal Child Psychology, 19, 553–567.

Deater-Deckard, K. (1996). The Parent Feelings Questionnaire. London: Institute of Psychiatry.

Derogatis, L. R. (1993). BSI, brief symptom inventory: Administration, scoring & procedures manual. National Computer Systems.

Fivush, R., Marin, K., McWilliams, K., & Bohanek, J. G. (2009). Family reminiscing style: Parent gender and emotional focus in relation to child well-being. Journal of Cognition & Development, 10(3). 210-235.

Frankenburg, W.K., et al., (1992). The Denver II: A major revision & restandardization of the Denver Developmental Screening Test. Pediatrics, 89(1), 91-97.

Frick, P. J., & Hare, R. D. (2001). Antisocial process screening device: APSD. Toronto: Multi-Health Systems.

Goodman, R. (1997). The Strengths and Difficulties Questionnaire: a research note. Journal of child psychology and psychiatry, 38(5), 581-586.

Holland, D. & M.R. Dadds, (1997). The DISCAP. 1997, Brisbane: Griffith University.

Hawes, D. J., Dadds, M. R., & Pasalich, D. (2013). Observational coding strategies. In J. Comer & P. Kendall (Eds.), The Oxford Handbook of Research Strategies for Clinical Psychology (pp.120-141). New York: Oxford University Press.

Matheny, A. P., Wachs, T. D., Ludwig, J. L., & Phillips, K. (1995). Bringing order out of chaos: Psychometric characteristics of the confusion, hubbub, and order scale. Journal of Applied Developmental Psychology, 16(3), 429-444.

Meins, E., & Fernyhough, C. (2015). Mind-mindedness coding manual, Version 2.2. Unpublished manuscript. University of York, York, UK.

Nauta, M. H., Scholing, A., Rapee, R. M., Abbott, M., Spence, S. H., Waters, A. (2004). A parent-report measure of children's anxiety: psychometric properties and comparison with child-report in a clinic and normal sample. Behaviour Research and Therapy , 42, 813–839.

Preacher, K.J. & Kelley, K. (2011). Effect size measures for mediation models. Psychological Methods, 16(2), 93-115.

Rey, J. M., Singh, M., Hung, S. F., Dossetor, D. R., Newman, L., Plapp, J. M., & Bird, K. D., (1997). A global scale to measure the quality of the family environment. *Archives of General Psychiatry, 54(9),* 817-822.

Runyan, D. K., Dunne, M. P., Zolotor, A. J., Madrid, B., Jain, D., Gerbaka, B., et al. (2009). The development and piloting of the ISPCAN Child Abuse Screening Tool-Parent version(ICAST-P). Child Abuse and Neglect. 33(11), 826–32.

Rutter M., Bailey A. & Lord C. (2003) Social Communication Questionnaire. Western Psychological Services, Los Angeles.

Spence, S. H. (1999). Spence Children's Anxiety Scale (parent version). Brisbane: University of Queensland.

Sanders, M. R., Morawska, A., Haslam, D. M. , Filus, A., & Fletcher, R. (2013). Parenting and Family Adjustment Scales (PAFAS): Validation of a Brief Parent-Report Measure for Use in Assessment of Parenting Skills and Family Relationships. Child Psychiatry and Human Development, 45(3), 255-272.
