# Supplementary material for: Using Time-out for Child Conduct Problems in the Context of Trauma and Adversity: A Nonrandomized Controlled Trial
Source: JAMA Netw Open. 2022 Sep 1;5(9):e2229726. doi: 10.1001/jamanetworkopen.2022.29726 (PMC9437765; doi:10.1001/jamanetworkopen.2022.29726)
Supplement: Supplement 2. — eFigure. CONSORT Flow Chart of the Study eTable 1. Repeated Measures ANOVA - Time x Condition, Time Period (T), Means (M), Standard Deviations (SD), Mean Differences Between Groups (MD), 95% Confidence Intervals (CI), and P Value (P) eTable 2. Repeated Measures ANOVA - Time x ACE Exposure, Time Period (T), Means (M), Standard Deviations (SD), Mean Differences Between Groups (MD), 95% Confidence Intervals (CI), and P Value (P) eTable 3. Repeated Measures ANOVA - Time x Maltreatment (MI) Exposure, Time Period (T), Means (M), Standard Deviations (SD), Mean Differences Between Groups (MD), 95% Confidence Intervals (CI), and P Value (P) [file jamanetwopen-e2229726-s002.pdf]

Supplementary Online Content

Roach AC, Lechowicz M, Yiu Y, Mendoza Diaz A, Hawes D, Dadds MR. Using time-out for child conduct problems in the context of trauma and adversity. *JAMA Netw Open.* 2022;5(9):e2229726. doi:10.1001/jamanetworkopen.2022.29726

eFigure. CONSORT Flow Chart of the Study
eTable 1. Repeated Measures ANOVA - Time x Condition, Time Period (T), Means (M), Standard Deviations (SD), Mean Differences Between Groups (MD), 95% Confidence Intervals (CI), and P Value (P)
eTable 2. Repeated Measures ANOVA - Time x ACE Exposure, Time Period (T), Means (M), Standard Deviations (SD), Mean Differences Between Groups (MD), 95% Confidence Intervals (CI), and P Value (P)
eTable 3. Repeated Measures ANOVA - Time x Maltreatment (MI) Exposure, Time Period (T), Means (M), Standard Deviations (SD), Mean Differences Between Groups (MD), 95% Confidence Intervals (CI), and P Value (P)

This supplementary material has been provided by the authors to give readers additional information about their work.

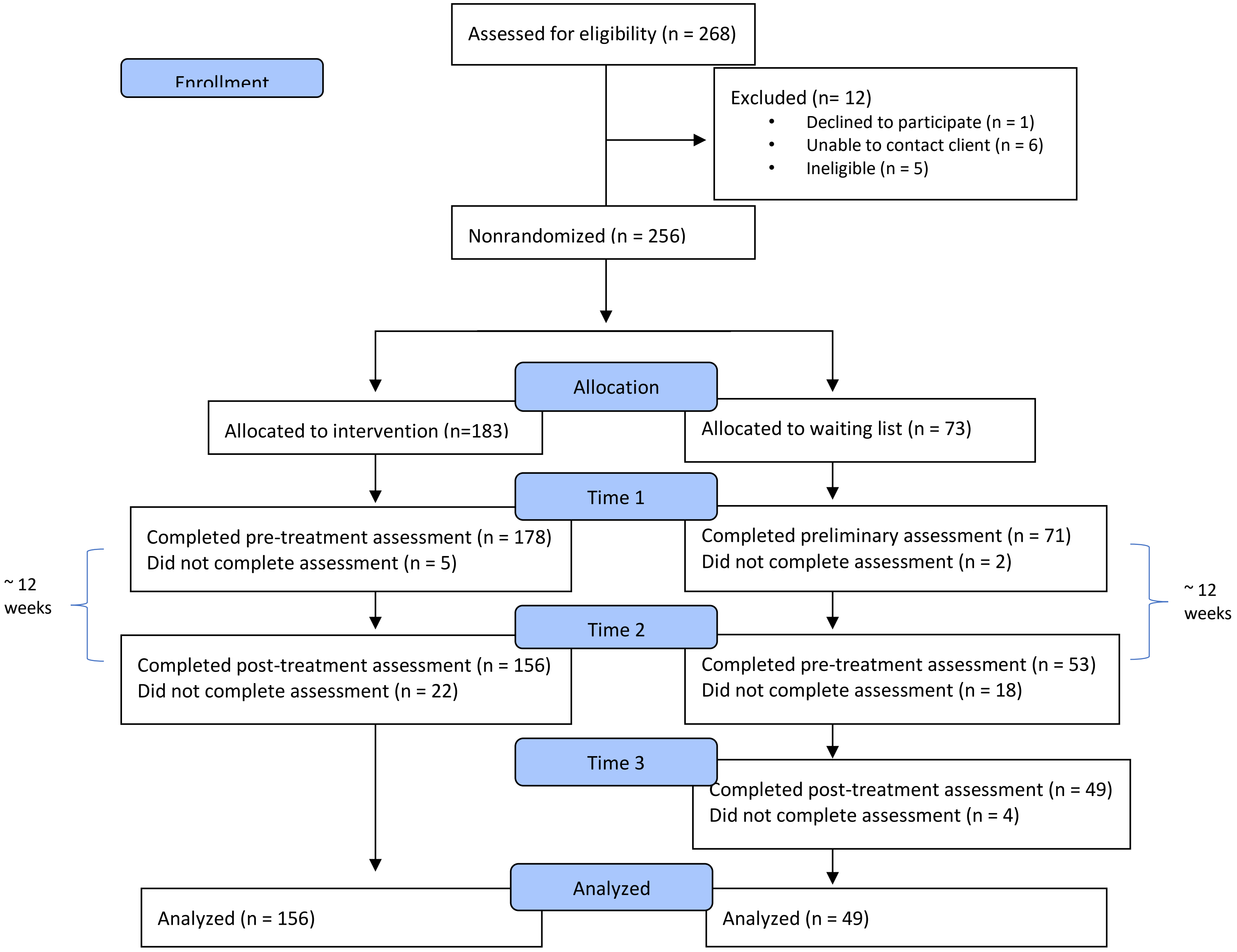

eFigure. CONSORT Flow Chart of the Study

© 2022 Roach AC et al. *JAMA Network Open.*

eTable 1. Repeated Measures ANOVA - Time x Condition, Time Period (T), Means (M), Standard Deviations (SD), Mean Differences Between Groups (MD), 95% Confidence Intervals (CI), and P Value (P)

	Condition							
	Waitlist (N = 49)		Intervention (N = 156)		Mean Difference Between Groups			
	**T1**	**T2**	**T1**	**T2**	**T1**		**T2**	
Outcomes	M(SD)	M(SD)	M(SD)	M(SD)	MD (95% CI)	*P*	MD (95% CI)	*P*
SDQ	18.44 (.73)	17.27 (.72)	17.65 (.40)	12.72 (.40)	.79 (-.86 – 2.45)	.35	4.55 (2.91 – 6.20)	<.001
Externalising DISCAP	4.04 (.13)	3.76 (.20)	3.99 (.07)	2.13 (.11)	.05 (-.25 - .35)	.74	1.63 (1.18 – 2.08)	<.001

© 2022 Roach AC et al. *JAMA Network Open.*

eTable 2. Repeated Measures ANOVA - Time x ACE Exposure, Time Period (T), Means (M), Standard Deviations (SD), Mean Differences Between Groups (MD), 95% Confidence Intervals (CI), and P Value (P)

	ACE Exposure							
	Low ACE (N = 122)		High ACE (N = 34)		Mean Difference Between Groups			
	**T1**	**T2**	**T1**	**T2**	**T1**		**T2**	
Outcomes	M(SD)	M(SD)	M(SD)	M(SD)	MD (95% CI)	*P*	MD (95% CI)	*P*
SDQ	17.18 (.44)	12.62 (.44)	20.64 (.86)	14.12 (.86)	3.46 (1.51 – 5.41)	<.001	1.49 (-.46 – 3.44)	.13
Externalising DISCAP	4.03 (.09)	2.14 (.14)	3.85 (.17)	2.13 (.26)	.18 (-.19 – .56)	.33	.01 (-.59 – .60)	.98
Internalising DISCAP	1.41 (.15)	.99 (.13)	1.50 (.28)	1.07 (2.5)	.10 (-.55 – .74)	.77	.08 (-.49 – .66)	.77

© 2022 Roach AC et al. *JAMA Network Open.*

eTable 3. Repeated Measures ANOVA - Time x Maltreatment (MI) Exposure, Time Period (T), Means (M), Standard Deviations (SD), Mean Differences Between Groups (MD), 95% Confidence Intervals (CI), and P Value (P)

	MI Exposure							
	Low MI (N = 137)		High MI (N = 11)		Mean Difference Between Groups			
	**T1**	**T2**	**T1**	**T2**	**T1**		**T2**	
Outcomes	M(SD)	M(SD)	M(SD)	M(SD)	MD (95% CI)	*P*	MD (95% CI)	*P*
SDQ	17.76 (.42)	12.96 (.41)	21.04 (1.53)	13.25 (1.46)	3.28 (.14 – 6.42)	.04	.29 (-2.72 – 3.30)	.85
Externalising DISCAP	3.98 (.08)	2.17 (.13)	4.26 (.28)	2.02 (.45)	.28 (-.29 – .85)	.33	.15 (-.78 – 1.08)	.76
Internalising DISCAP	1.33 (.13)	.96 (.19)	2.32 (.48)	1.01 (.43)	1.00 (-2.00 – .00)	.05	.06 (-.82 – .94)	.90

© 2022 Roach AC et al. *JAMA Network Open.*
