# Supplementary material for: Using Time-out for Child Conduct Problems in the Context of Trauma and Adversity: A Nonrandomized Controlled Trial
Source: JAMA Netw Open. 2022 Sep 1;5(9):e2229726. doi: 10.1001/jamanetworkopen.2022.29726 (PMC9437765; doi:10.1001/jamanetworkopen.2022.29726)
Supplement: Supplement 3. — Data Sharing Statement [file jamanetwopen-e2229726-s003.pdf]

Data Sharing Statement

Roach. Using Time-Out for Child Conduct Problems in the Context of Trauma and Adversity. *JAMA Netw Open*. Published September 01, 2022. doi:10.1001/jamanetworkopen.2022.29726

Data
Data available: Yes
Data types: Deidentified participant data
How to access data: mark.dadds@sydney.edu.au
When available: With publication

Supporting Documents
Document types: None

Additional Information
Who can access the data: Researchers whose proposed use of the data has been approved.
Types of analyses: For specified purposes.
Mechanisms of data availability: After approval of a proposal.
